# Cardiac Rhythm Monitoring Using Wearables for Clinical Guidance before and after Catheter Ablation

**DOI:** 10.3390/jcm11092428

**Published:** 2022-04-26

**Authors:** Henrike Aenne Katrin Hillmann, Samira Soltani, Johanna Mueller-Leisse, Stephan Hohmann, David Duncker

**Affiliations:** Hannover Heart Rhythm Center, Department of Cardiology and Angiology, Hannover Medical School, 30625 Hannover, Germany; hillmann.henrike@mh-hannover.de (H.A.K.H.); soltani.samira@mh-hannover.de (S.S.); mueller-leisse.johanna@mh-hannover.de (J.M.-L.); hohmann.stephan@mh-hannover.de (S.H.)

**Keywords:** mHealth, arrhythmia, cardiac monitoring, remote monitoring, digital health, wearable, telemonitoring, electrophysiological study, catheter ablation, atrial fibrillation

## Abstract

Mobile health technologies are gaining importance in clinical decision-making. With the capability to monitor the patient’s heart rhythm, they have the potential to reduce the time to confirm a diagnosis and therefore are useful in patients eligible for screening of atrial fibrillation as well as in patients with symptoms without documented symptom rhythm correlation. Such is crucial to enable an adequate arrhythmia management including the possibility of a catheter ablation. After ablation, wearables can help to search for recurrences, in symptomatic as well as in asymptomatic patients. Furthermore, those devices can be used to search for concomitant arrhythmias and have the potential to help improving the short- and long-term patient management. The type of wearable as well as the adequate technology has to be chosen carefully for every situation and every individual patient, keeping different aspects in mind. This review aims to describe and to elaborate a potential workflow for the role of wearables for cardiac rhythm monitoring regarding detection and management of arrhythmias before and after cardiac electrophysiological procedures.

## 1. Introduction

Mobile health (mHealth) technologies have gained an important role, not only for lifestyle purposes, but also in clinical decision-making. Among others, they can be used to screen for arrhythmias and to establish a diagnosis. Different wearables including varying capabilities have been developed over time. Screening, documentation and confirmation of arrhythmias may necessitate another type of technology than searching for arrhythmia recurrences after an electrophysiological procedure. Therefore, finding the best technology for the individual case and patient is crucial. Several reviews have illuminated the role of wearables in screening of arrhythmias, mostly for atrial fibrillation (AF) [1,2,3,4] but also for other types of arrhythmias beyond AF [3,5]. In this review, we aim to describe the potential role of wearables regarding detection and management of arrhythmias before and after cardiac electrophysiological procedures and to elaborate a potential workflow.

## 2. Clinical Use of Wearables

**Figure 1:**
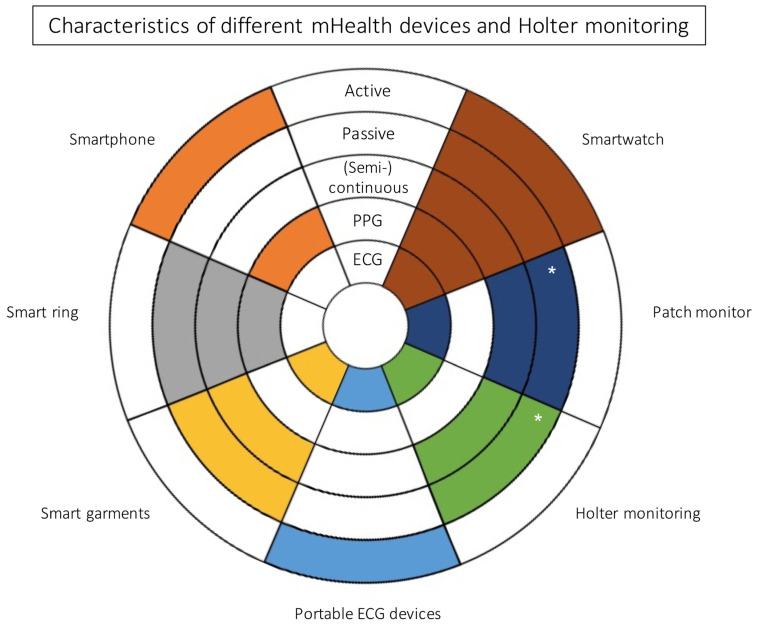
Characteristics of different mHealth devices and Holter monitoring. Colored fields signify that the wearable has the capability described. “Active” regards to the patients’ possibility of manually record an episode, whereas “passive” refers to monitoring via automatically recorded episodes using the individual device. A (semi-)continuous tracking describes the ability of the device to monitor patients’ heart rhythm via automatic pre-set intervals. PPG = photoplethysmography; ECG = electrocardiogram; * for a limited time.

Multiple types of mHealth devices with the capability of monitoring heart rate and detection of arrhythmias have been developed [6,7]. Smartwatches, such as the Apple Watch (Apple Inc., Cupertino, CA, USA) and portable ECG devices such as KardiaMobile (AliveCor Inc., Mountain View, CA, USA) are best known [8], but other less popular tools, such as electrocardiography patches, chest belts, t-shirts, glasses or rings, may also be used for arrhythmia monitoring [1,9]. Wearables vary from mHealth devices with additional hardware, such as external electrodes, to mHealth devices with integrated electrodes, such as smartwatches [9]. Different mHealth devices have different capabilities of monitoring and recording (Figure 1).

Irrespective of the intended use, one should take into consideration that many wearables do not have a medical validation. The recommendation is to only use validated mHealth solutions for diagnostic and therapeutic approach [9].

Regarding the use of wearables in a clinical setting, two technologies are known for identification of heart rate and detection of cardiac arrhythmias [9]: photoplethysmography (PPG) and electrocardiogram (ECG)-based technology. The PPG is based on light absorption and pulsatile reflection of capillaries [10]. ECG-based wearables provide single-lead to multiple lead ECG [9].

## 3. Confirmation of Diagnosis

Until today, cardiac rhythm monitoring is known to be useful especially in two situations: to screen for AF as well as to target a symptom rhythm correlation in patients suffering from symptoms assumed to be related to arrhythmias.

According to current guidelines, opportunistic screening for AF is recommended in patients ≥ 65 years [11]. Early detection of AF is useful to reduce the risk of stroke by an early start of an oral anticoagulation, on the one hand, and to reduce AF-related comorbidities and outcomes [11]. In addition to the early start of a therapy regarding the rate control, an early rhythm control has been found not only to reduce symptoms, but also to delay the progress of AF [12] and therefore to be prognostically relevant [13]. Since prognostic relevance is important in patients having AF even without symptoms [14], early detection is useful and can lead to the establishment of an early and structured integrated AF management. In this matter catheter ablation is one possible and relevant treatment option.

In patients with symptoms, early establishment of a diagnosis is important: non-cardiac symptom causes should be ruled out and possible treatment options, such as a catheter ablation, may be offered depending on the diagnosis and requiring a symptom rhythm correlation.

In the past, tools such as intermittent ECG rhythm strips as well as 24 h Holter monitoring were used to correlate symptoms as well as to search for AF. However, those tools have the disadvantage that establishment of a diagnosis may be cumbersome, especially in patients with paroxysmal atrial fibrillation, rare episodes or infrequent symptoms. The addition of wearables in the process of searching for AF or other arrhythmias may help to reduce the time to diagnosis. Advantages and disadvantages of wearables in difference to traditional tools are shown in Table 1.

**Figure 2:**
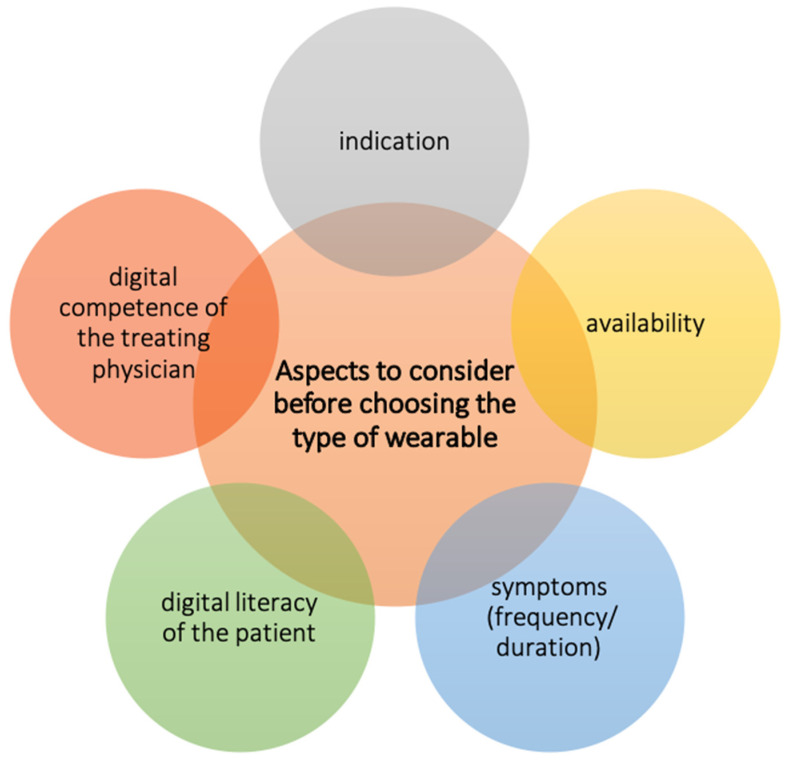
Aspects to consider before choosing the type of wearable.

As different wearables vary in availability, recording capability and technology, they have to be chosen carefully. Important aspects to be taken into consideration should be the indication, availability, digital literacy as well as frequency and duration of symptoms (Figure 2) [15].

Active recording methods may be reasonable in patients with symptoms, whereas passive recording methods may be more suitable in asymptomatic patients while searching for AF. Nevertheless, symptomatic patients with less digital competence may benefit from passive (semi-)continuously recording methods such as ECG patches or wearable belts as often being easier to handle.

In patients with infrequent symptoms, wearables with a limited recording time (e.g., patches) may not be sufficient to confirm a diagnosis. Therefore, wearables with an unlimited recording time may be preferred.

PPG- and ECG-based technologies can be used to primarily screen or to target a symptom rhythm correlation, not only for AF [1,16,17] but also for arrhythmias other than AF [5,18]. Nevertheless, a physician-confirmed 12-lead ECG or 30 s of a single-lead ECG is mandatory to confirm the diagnosis of atrial fibrillation [11] as well as to confirm the diagnosis of arrhythmias other than AF.

Whereas a multiple lead ECG may be beneficial to identify the mechanism of different tachycardias such as atrioventricular nodal reentry tachycardia (AVNRT), atrioventricular reentry tachycardia (AVRT), atrial flutter or atrial tachycardias, a single-lead ECG may be sufficient to confirm the indication for an electrophysiological study. Nevertheless, studies have shown that single-lead ECGs can be modified to use as a multiple lead ECG due to multiple recordings [19,20,21,22]. Case series and studies evaluating the use of wearables before and after electrophysiological procedure for cardiac arrhythmias are summarized in Table 2.

## 4. Monitoring for Recurrences and Concomitant Arrhythmias

Rhythm monitoring after catheter ablation of arrhythmias is useful to identify recurrences as well as to search for concomitant arrhythmias. Current guidelines recommend rhythm monitoring after an AF ablation to assess the ablation success [11]. As recurrence of AF beyond the first month after catheter ablation is known to be predictive for late recurrences [27,28], the identification of early recurrences as well as knowledge of the burden gives the physician the opportunity to promptly react to the patient needs. This enables improvement of short- and long-term rhythm management after AF ablation [27,28,29], not only in symptomatic but also in asymptomatic patients. Moreover, the doctor–patient relationship may be intensified and therefore improved. ECG-based, but also PPG-based technologies have been shown to be adequate to search for recurrences and therefore improve the long-term patient management [30,31,32]. In the TeleCheck-AF project developed during COVID-19 [31,32,33], in which several centers included patients, e.g., prior as well as post electrical cardioversion or AF ablation, most centers stated that mHealth approaches will be used for follow-up after an AF ablation in the future [16]. To identify recurrences of known arrhythmias, PPG technology or ECG technology may both be used in patients after ablation of AF. However, only an ECG will allow diagnosis of new arrhythmias.

Monitoring of patients with arrhythmias other than AF might not be necessary after every procedure, but might be useful for patients with recurrent symptoms to target a symptom rhythm correlation on the one hand and to search for concomitant arrhythmias after an ablation on the other hand. Atrial flutter is known to be associated with AF [34]. Therefore, it might be useful to monitor patients after ablation of atrial flutter to rule out this concomitant arrhythmia for improvement of individual patient management. Knowing the cycle length of the treated arrhythmia documented during the ablation procedure may help to interpret PPG- as well as ECG-based recordings after catheter ablation and to differentiate recurrences from concomitant arrhythmias, not only for AF but also for arrhythmias other than AF [5]. To search for AF after ablation of atrial flutter or in case of recurrent symptoms, PPG-based technologies can be used, but, as mentioned before, an ECG is mandatory to confirm the diagnosis of AF or arrhythmias other than AF.

**Figure 3:**
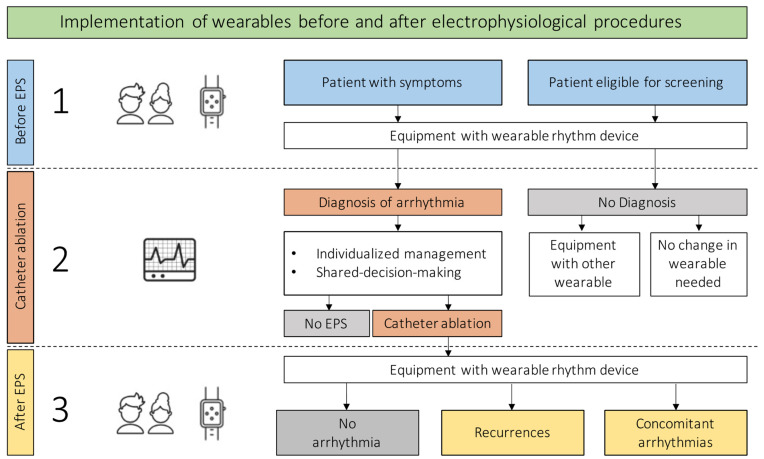
Implementation of wearables for cardiac rhythm monitoring before and after electrophysiological procedures. EPS = electrophysiological study.

A possible workflow regarding the implementation of wearables to confirm the diagnosis as well as to search for recurrences or concomitant arrhythmias after catheter ablation is shown in Figure 3.

**Figure 4:**
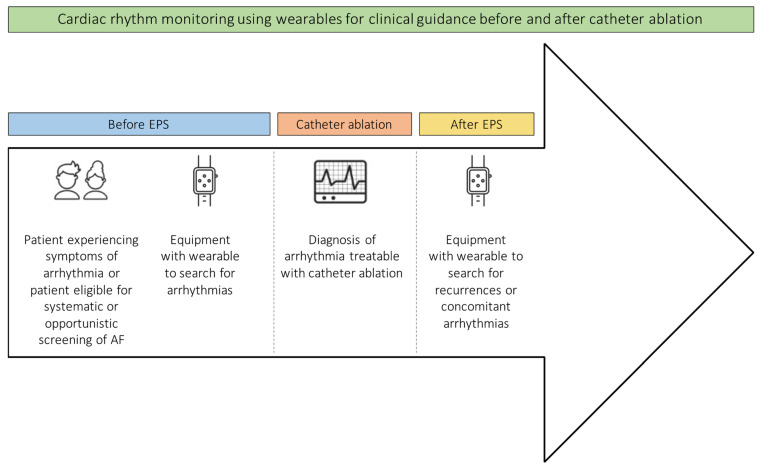
Cardiac rhythm monitoring using wearables for clinical guidance before and after catheter ablation. EPS = electrophysiological study; AF = atrial fibrillation.

In patients using wearables, recurrences may be found easier than in patients using routine methods such as 24-h Holter ECGs or 12-lead ECGs and are less invasive and mostly cheaper than implantable loop recorders. Having the possibility of remote monitoring, patient management often can be improved due to close monitoring (Figure 4).

If mobile health technologies fail to provide relevant information regarding the detection of arrhythmias, the physician should question the reason for this issue, verifying, e.g., the digital competence of the patient as well as the frequency of symptoms. As stated within a recently published EHRA practical guide on the use of digital devices to detect and manage arrhythmias, commonly known tools can be taken into consideration as well—for daily or monthly symptoms, a Holter ECG can be used as an alternative for a wearable; for yearly symptoms an implantable loop recorder may be taken into consideration [15]. Nevertheless, mobile health technologies advance rapidly increasing the possibilities in the diagnostic of arrhythmias. Therefore, the development of such different technologies should be observed carefully. 

ECG- and PPG-based recordings should always be validated by the attending physician to confirm a diagnosis. Therefore, adequate interpretation of all recordings is crucial. As the interpretation of PPG-based tracings can be challenging for the attending physicians [5,8,35], physicians should be trained on how to use PPG tracings before using this relatively new technology. Regarding training, a possible workflow [36] as well as a how-to guide [10,37] have been published recently.

## 5. Conclusions

Early confirmation of a diagnosis is crucial for an adequate arrhythmia management. Wearables may shorten time to diagnosis and therefore help to establish a prompt and structured arrhythmia management including the necessity of an electrophysiological procedure. After catheter ablation, wearables can help to search for recurrences or concomitant arrhythmias and therefore help to establish and to improve an adequate short- and long-term management. The appropriate type of wearable has to be chosen carefully for the individual patient taking different aspects into account. PPG-based wearables may help to recognize abnormalities or recurrences after catheter ablation, but an ECG will be needed to confirm the diagnosis.

## Figures and Tables

**Table 1:** Advantages and disadvantages of wearable cardiac rhythm devices.

Advantages	Disadvantages
May reduce time to diagnosis	Can imply costs for the patient
High availability	Lack of reimbursement for the treating physician
Possibility to improve arrhythmia management	Data overload
Remote monitoring option	Limited experience of physicians

**Table 2:** Case series and studies evaluating the use of wearables before or after catheter ablation of arrhythmias. PPG = photoplethysmography; ECG = electrocardiogram; FDA = Food and Drug administration; AVNRT = atrioventricular nodal reentry tachycardia; AVRT = atrioventricular reentry tachycardia; AF = atrial fibrillation. * The patient recorded tachycardias with two different cycle lengths (tachycardia 1 at 400–374 ms and palpitations; tachycardia 2 at 333 ms and syncope).

Author	Device (Specification)	Technology	FDA Approved	Number of Patients	Timing	Arrhythmia Ablated
Kasai et al. [23]	Smartwatch (Apple Watch SE)	PPG (heart rate)	Yes	1	Preprocedural	AVNRT, AVRT *
Siddeek et al. [24]	Smartwatch (Apple Watch Series 4)	ECG (single-lead)	Yes	1	Preprocedural	AVNRT
Wu et al. [25]	Smartwatch (Apple Watch—no detailed differentiation)	ECG (single-lead)	Yes	3	Preprocedural	AVRT, AVNRT
Aljuaid et al. [26]	Portable heart monitor (ECG check cardiac designs)	ECG (single-lead)	Yes	45	Postprocedural after AF ablation	-
